# Synchronous Motor with Hybrid Permanent Magnets on the Rotor

**DOI:** 10.3390/s140712425

**Published:** 2014-07-10

**Authors:** Barbara Slusarek, Dariusz Kapelski, Ludwik Antal, Pawel Zalas, Maciej Gwoździewicz

**Affiliations:** 1 Tele and Radio Research Institute, 11 Ratuszowa St., 03-450 Warsaw, Poland; E-Mail: dariusz.kapelski@itr.org.pl; 2 Wroclaw University of Technology, 27 Wybrzeze Wyspianskiego St., 50-370 Wroclaw, Poland; E-Mails: ludwik.antal@pwr.edu.pl (L.A.); pawel.zalas@pwr.edu.pl (P.Z.); maciej.gwozdziewicz@pwr.edu.pl (M.G.)

**Keywords:** magnet system, soft magnetic materials, electric motors

## Abstract

Powder metallurgy allows designers of electric motors to implement new magnetic circuit structures. A relatively new concept is the use of a magnet system consisting of various types of magnets on one rotor, for example sintered and bonded magnets. This concept has been applied to the design and manufacture of the four-pole rotor of a synchronous motor with 400 W power and a rotational speed of 1500 rpm. In this motor, the stator of an asynchronous motor type Sh 71-4B is applied. The application of the new construction of the rotor resulted in an increase in motor efficiency and power factor compared to an asynchronous motor with the same volume.

## 1. Introduction

Hard and soft powder magnetic materials have numerous advantages, and therefore they are finding more and more applications in magnetic circuits for electric machines. In particular, one of the methods of powder metallurgy, bonding powder by resin, makes it possible to prepare magnetic circuit elements with the physical properties required by designers of electric machines. The production technology allows the production of elements of net shape and size with high accuracy and without additional machining [1,2]. Sintered and bonded neodymium magnets have been on the market for many years. In the latter case, bonded magnets are characterized by worse magnetic properties, but are easier to manufacture. The solution of combining stronger and weaker magnets in a magnetic circuit is one of the areas of use of bonded magnets developed in recent years. The work presents the construction and realization technology of a compact rotor with hybrid permanent magnets for use in low power synchronous motor, designed to synchronous starting [3] and drive pumps and fans.

## 2. FEM Simulation

**Figure 1.**
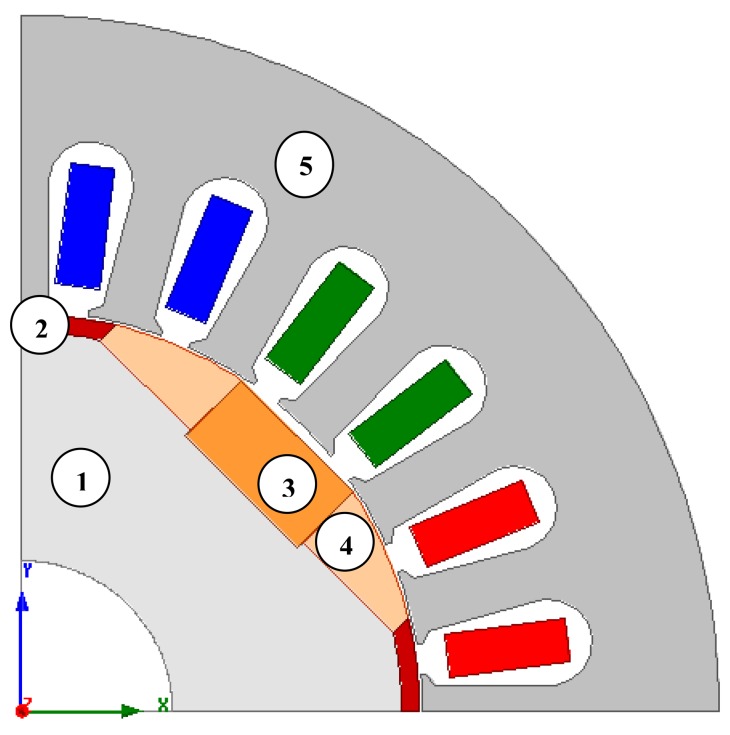
2D FEM model of permanent magnet synchronous motor with copper cage and hybrid magnets 1: soft magnetic composite core, 2: copper cage, 3: Sm-Co magnet, 4:Nd-Fe-B bonded magnet, 5: electrical sheets of the stator.

Several 2D simulation analyses were carried out. Maxwell version 14.0 software was used and a field model of the structure of a motor was created. Figure 1 shows the model used in the simulation studies. Characteristics of the materials used in the model and prototype were similar and are described later in the article. The studies focused on analyses of the starting of an electric motor and the calculation of its cogging torque. Calculations were conducted for different types of supply and motor loads. The method of supply is essential for starting the motor, but the type of load torque is important for both the starting and the synchronization of the motor. In the simulation analyses, the inertia of the drive system was also included.

**Figure 2.**
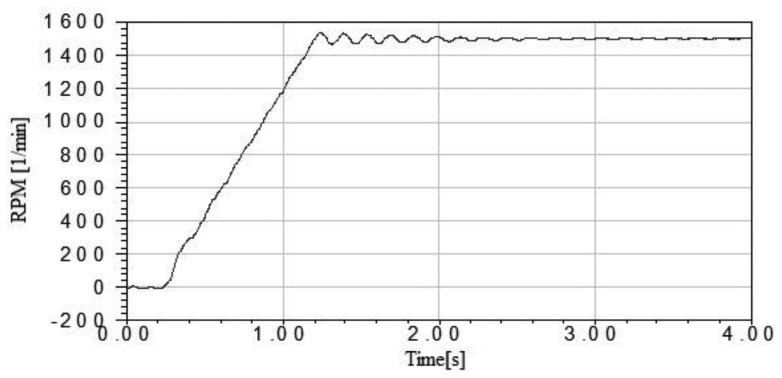
The characteristic of rotational speed during starting.

The synchronous starting of the motor with a pulse-width modulation (PWM) inverter was executed with adjustable amplitude and frequency of the voltage. In the simulations, the initial winding was supplied with a DC voltage, so that the rotor moved into the best position for starting. The starting was realized with a voltage equal to 15% of rated voltage. During the starting, the voltage and frequency increased linearly. After the rated speed was obtained, the phenomenon of a change in the rotational speed occurred. Whether these oscillations are dampened depends on the nature of the load torque. If the load torque depends on the speed of the vibration, the oscillations can be suppressed. The motor loading was simulated using Maxwell software. The fan type of load torque characteristic was used in the calculation [4–6]. The model of a motor also takes into account the value of inertia of the drive system. The simulation results showed that synchronization of the motor is possible, but the quality depends on the type of loading. Figure 2 shows the characteristics of the rotational speed of a rotor as a function of time.

**Figure 3.**
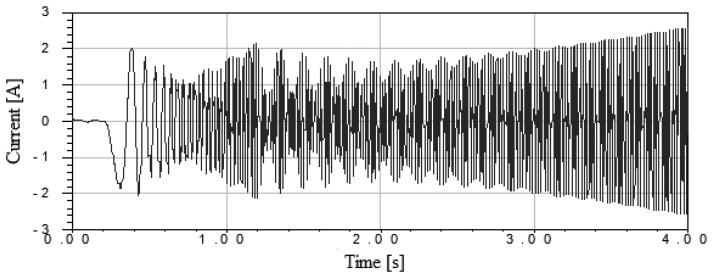
The characteristic of current during starting and the motor overload.

The presented structure is suitable for a fan drive whose speed is controlled by the inverter. The next stage of the simulation was the determination of the motor overload. For this purpose, the load torque of the motor was increased from 2 to 4 s. Figure 3 shows the current characteristic as a function of time with increasing load torque. It was proven in simulations that it is possible for the motor to work with a linear increase of the load torque to twice the rated torque [7,8].

**Figure 4.**
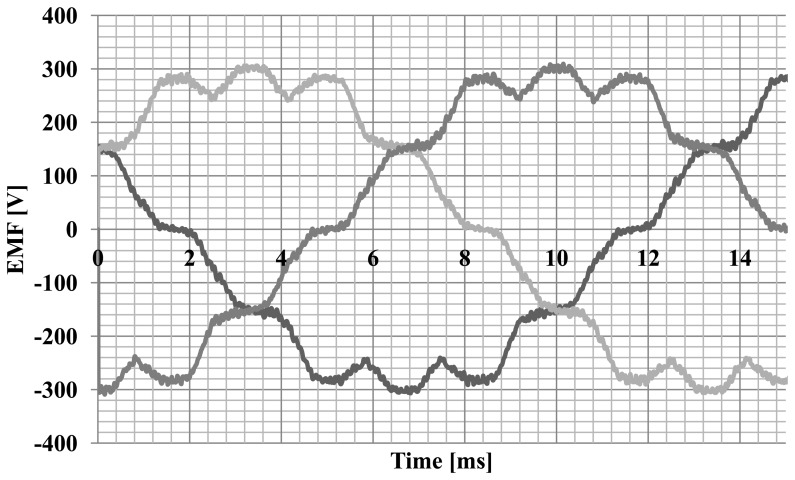
The characteristic of the electromotive force.

**Figure 5.**
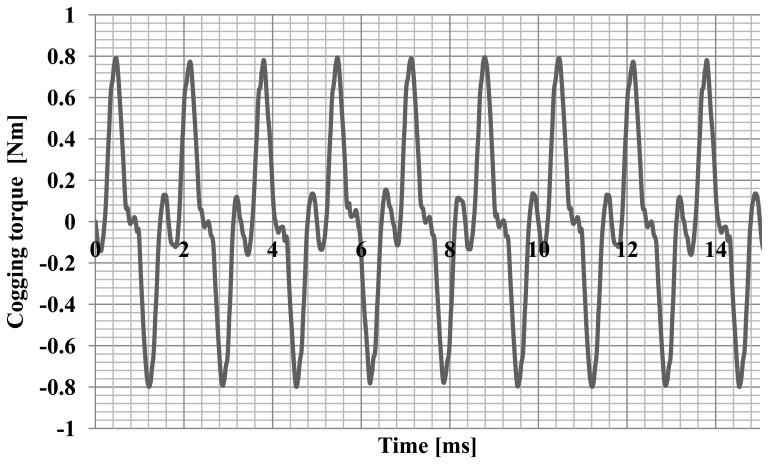
The characteristic of the cogging torque.

Simulation studies of machine operating parameters were carried out. The characteristics of the electromotive forces (Figure 4) generated by the hybrid magnet system and the characteristics of the cogging torque (Figure 5) were calculated.

**Figure 6.**
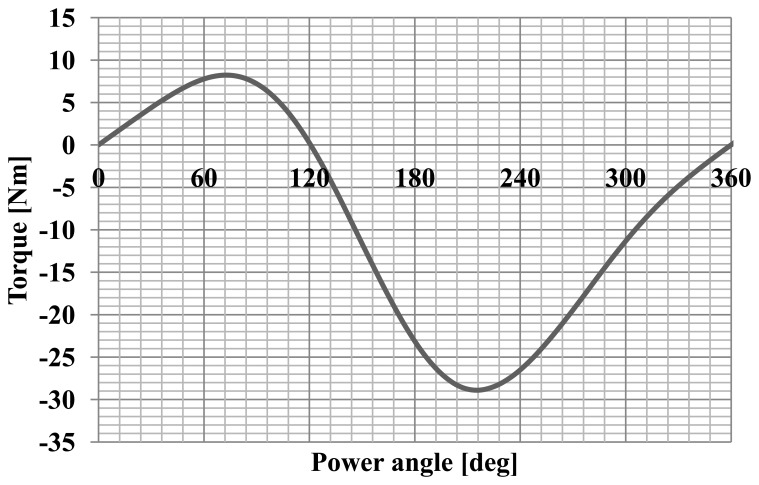
The characteristics of torque as a function of the power angle.

**Figure 7.**
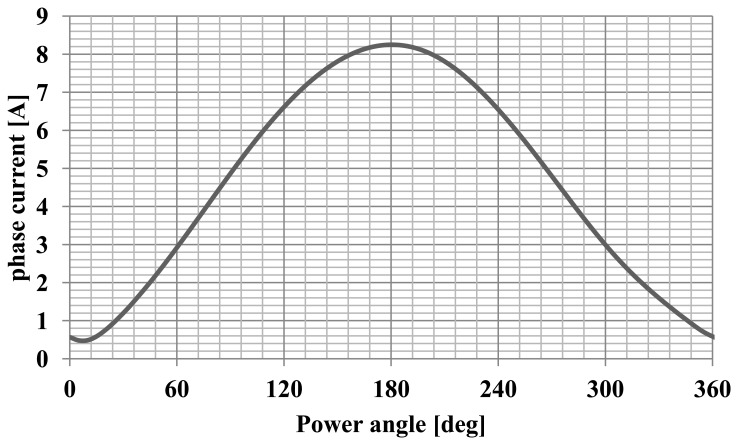
The characteristics of phase current as a function of the power angle.

The maximum of the cogging torque was 0.8 Nm, which is 30% of the nominal torque. Furthermore, the angular characteristics of the torque and current were calculated. Successful synchronization of synchronous motor at a load means the adoption by the rotor a particular, angular position relative to rotating stator magnetic field. The calculation of such characteristics allow to determine the maximum torque, maximum and rated load and static overload capacity the motor for a specific value and frequency of voltages. Numerical calculations were performed using the developed field-circuit model of computing for the dependent state of synchronous operation. This model has allowed to determine the torque and the current drawn by the motor as a function of the angle of the power. The change in motor load was achieved by changing the position of the axis of the rotor field relative to the axis of the rotating stator field. The motor was supply with the rated value and frequency of voltages what allows computing the angular-torque characteristic and angular-current characteristic. Diagrams of these characteristics for a synchronous motor model are shown in Figures 6 and 7, and the waveforms' analysed values are typical for this type of machine [9].

**Figure 8.**
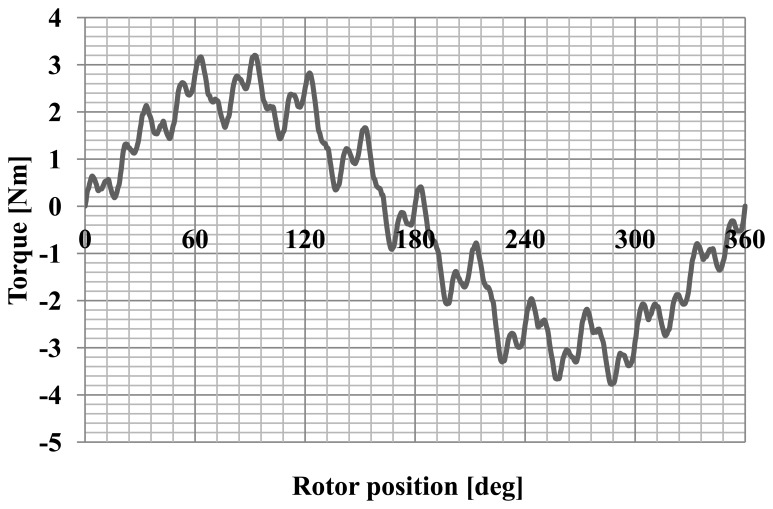
The characteristic of the static torque.

The static characteristics of the motor were determined. The torque as a function of the angle of rotation of the rotor was calculated for RMS phase current of 1A. The static characteristic of the motor is shown in Figure 8.

In addition, the simulations carried out checking out the possibility of direct starting. In simulations the motor was supplied with constant voltage and frequency and was loaded with fan type torque with nominal values. The simulation results show that the motor cannot achieve synchronization state and gets stuck at an average speed of about 80 rpm. The analyses of the simulation results thus indicate that direct starting (with constant voltage and frequency) of the motor is not possible, regardless of the nature and value of the load torque. The rotor cage placed between hybrid magnets, with dimensions which result from the assumptions of hybrid technology, does not enable asynchronous starting. Asynchronous torque generated by cage during starting is smaller than the cogging torque. Therefore, a copper cage was used for damping oscillations of the rotor speed and for positioning the magnets during installation.

## 3. Construction of Rotors with Hybrid Magnets Based on Powder Metallurgy

**Figure 9.**
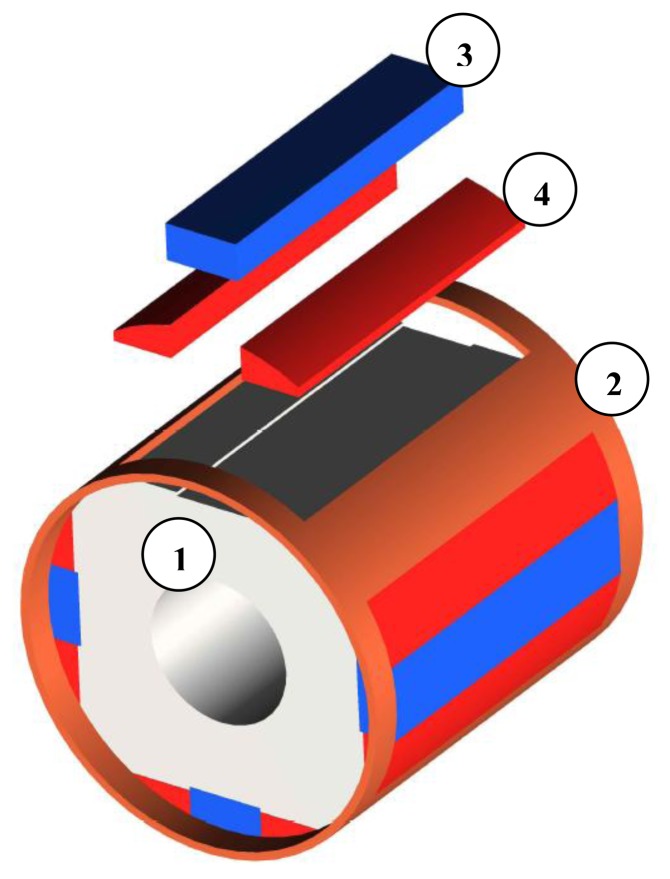
The designed rotor: 1: Soft magnetic composite core, 2: Copper cage, 3: Sm-Co magnet, 4: Nd-Fe-B bonded magnet, packet length: 56 mm.

Hybrid magnets are systems consisting of magnets having different properties and are used to achieve the desired distribution of magnetic induction in the air gap of electric motors. Designing rotors using such solutions requires the use of the finite element method. The proposed solution is to use bonded Nd-Fe-B magnets and Sm-Co magnets. The cross-section and a model of the rotor are shown in Figure 9.

**Figure 10.**
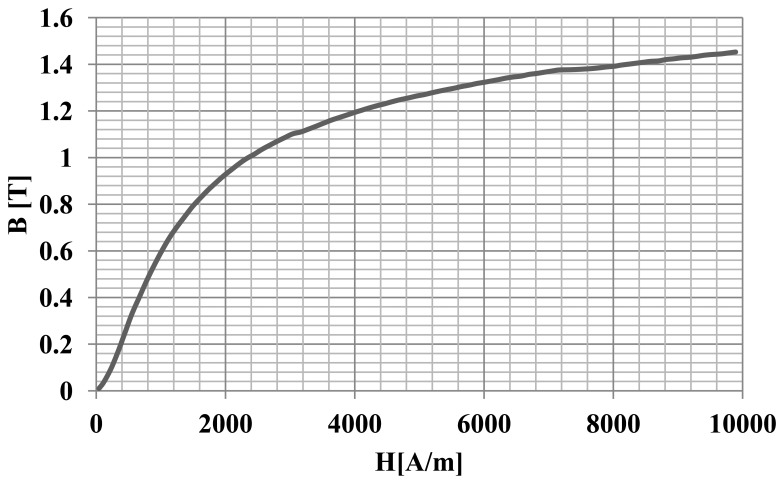
Characteristic of magnetization of Somaloy 700.

Two kinds of magnets were used for the construction of the rotor. First, bonded permanent magnets were made of Nd-Fe-B melt-spun ribbon powder. Then, 2.5 wt% of epoxy resin (Epidian 100) and 0.2 wt% of zinc stearate, as a lubricant, were added to the powder mixture [1]. Magnets were pressed at a pressure of 900 MPa and then cured in a laboratory oven at 180 °C for two hours. Bonded magnets had the following magnetic characteristics: B_r_ = 0.64 T, H_cJ_ = 621 kA/m, H_cB_ = 394 kA/m. The second kind of magnet was an Sm-Co sintered magnet, type S30. The rotor core was made from a soft magnetic composite made of a powder called Somaloy 700 [2]. Figure 10 shows characteristics of magnetization the of the soft magnetic composite Somaloy 700 (used in the simulation).

**Figure 11.**
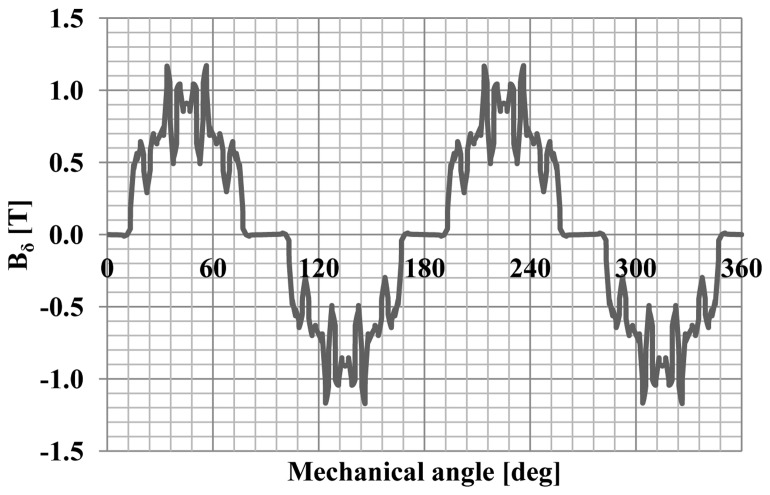
Distribution of flux density in air gap of motor.

Mounting a hybrid system on the surface of the rotor magnets is difficult, because the magnets must be placed side by side with the same direction of magnetization. In order to construct the prototype motor, it was necessary to develop a method of mounting the magnets on the rotor surface. The copper cage allowed to determine the position of the magnets, and has facilitated their installation. Such a solution should allow for modelling of the distribution of the flux density in the gap of an electric machine. Figure 11 shows a distribution of the flux density in the gap obtained by the use of the hybrid magnets.

For tests, the stator of an asynchronous motor of type Sh 71-4B was used.

## 4. Testing of Motor Prototype

The research was conducted to compare the functional parameters of the prototype motor and an asynchronous motor with a squirrel-cage rotor of type SH 71-4B [10]. The parameters of both motors and the measurement results are shown in Table 1. The results summarized in Table 1 indicate that the application of the hybrid rotor resulted in an increase in the motor efficiency from 63% to 78% and an increase in the power factor from 0.68 to 0.97.

**Figure 12.**
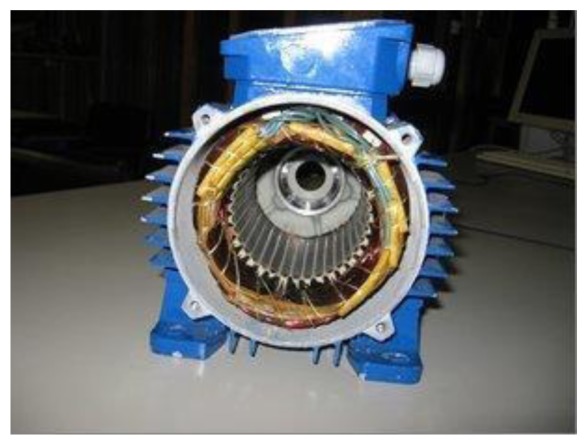
The stator of the Sh 71-4B motor.

**Figure 13.**
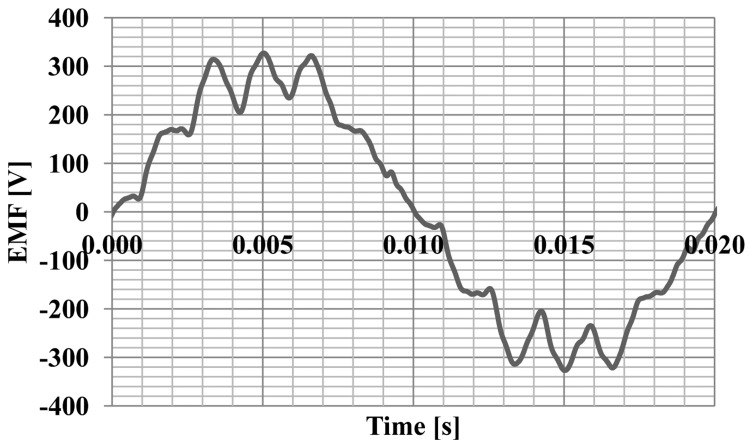
EMF characteristic of the motor in a steady state at a speed of 1500 rpm.

In addition, measurements of EMF induced in the stator windings were conducted. In this test, an external motor was used to drive the rotor with hybrid magnets to 1500 rpm. During the tests the phase currents were also measured at nominal load. The graphs are shown in Figure 12. The EMF illustrated in Figure 13 has a trapezoidal shape and the current waveform is close to sine.

**Figure 14.**
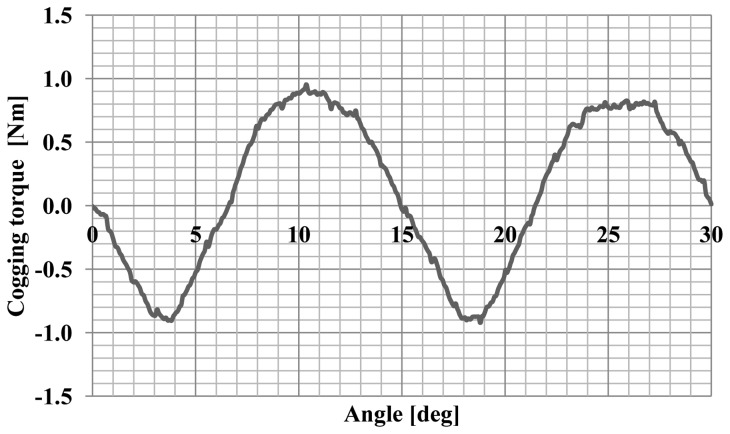
The cogging torque characteristic of the motor.

**Figure 15.**
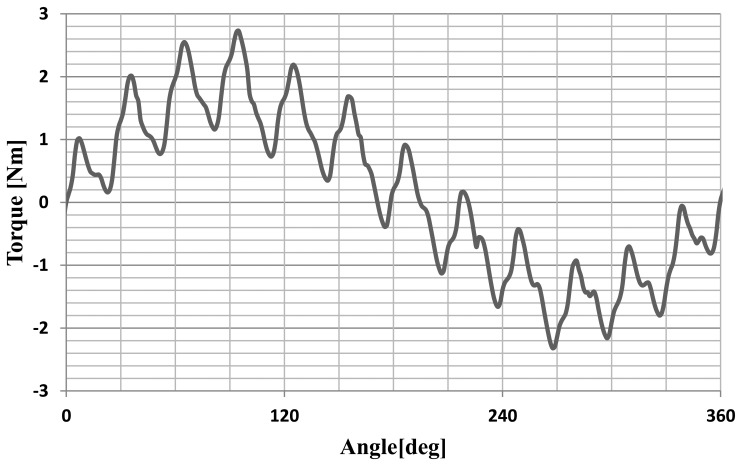
The static torque characteristic of the motor for a current of 1A.

The motor cogging torque characteristic was also measured as a function of the angle of rotation. The cogging torque characteristic is shown in Figure 14. Figure 15 shows the static torque characteristic for the RMS phase current of 1A.

The cogging torque of the motor is about 30% of the rated torque. This is a very large value. A large cogging torque influences the oscillatory nature of the static torque characteristics. Reduction of this phenomenon can be achieved by the use of magnetic wedges.

## 5. Conclusions

Simulation analysis shows that direct starting is not possible for the analysed motor. Starting is possible only with an electronic inverter. Using Maxwell software, the overload of the motor was calculated. Applications of hybrid magnets show that it is possible to increase cos φ of the motor and to reduce the cogging torque.

Initial measurements of the prototype motor show that the use of a combined magnet system allows for satisfactory performance of the drive. The solution makes it possible to obtain about 24% higher efficiency than in the case of an asynchronous motor. The use of two different types of magnets in one motor is not an easy solution, but it allows the distribution of the magnetic flux density in the gap to be modelled and leads to increased efficiency. The application of iron composite for the construction of the rotor does not negatively affect the motor parameters, and could also allow the cost of production of such rotors to be reduced. The use of the hybrid magnet system does not reduce the cogging torque in the motor, just as originally expected. Work on the use of more complex structures of hybrid magnets will be continued in the future.

The results of simulations and prototype measurements are characterized by a small difference. One of reasons is the influence of the machining process on parameters of composite magnetic materials. Mechanical processing was necessary during the construction of the prototype. In addition, the finite element method used the catalog data of the characteristics of Sm-Co permanent magnets. The model does not take into account the inaccuracy of machining the rotor and the occurrence of air gaps between the magnets. In addition, the rotor has not been well balanced before testing.

## Figures and Tables

**Table 1.** Parameters of electric motor with hybrid magnet system and asynchronous motor.

	**Voltage V**	**Power W**	**Speed rpm**	**Current A**	**Efficiency %**	**cos φ [−] −**	**Torque Nm**
Asynchronous motor Sh 71-4B	400	370	1370	1.25	63	0.68	2.59
**Motor with hybrid magnet system**	**275**	**400**	**1500**	**1.11**	**78**	**0.97**	**2.57**
